# The crystal structure of CbpD clarifies substrate-specificity motifs in chitin-active lytic polysaccharide monooxygenases

**DOI:** 10.1107/S2059798322007033

**Published:** 2022-07-27

**Authors:** Christopher M. Dade, Badreddine Douzi, Christian Cambillau, Genevieve Ball, Romé Voulhoux, Katrina T. Forest

**Affiliations:** a Department of Chemistry, University of Wisconsin-Madison, Madison, Wisconsin, USA; b Aix-Marseille University, CNRS, IMM, LCB, Marseille, France; c Aix-Marseille University, CNRS, AFMB, Marseille, France; d Department of Bacteriology, University of Wisconsin-Madison, Madison, Wisconsin, USA

**Keywords:** LPMOs, lytic polysaccharide monooxygenases, AA10, *Pseudomonas aeruginosa*, chitin binding protein CbpD, type 2 secretion systems, *RoseTTAFold*, biofuels, bacterial pathogenesis

## Abstract

*Pseudomonas aeruginosa* secretes diverse proteins via its type 2 secretion system, including a 39 kDa chitin-binding protein, CbpD. CbpD has recently been shown to be a lytic polysaccharide monooxygenase active on chitin and to contribute substantially to virulence. To date, no structure of this virulence factor has been reported. Its first two domains are homologous to those found in the crystal structure of *Vibrio cholerae* GbpA, while the third domain is homologous to the NMR structure of the CBM73 domain of *Cellvibrio japonicus* *Cj*LPMO10A. Here, the 3.0 Å resolution crystal structure of CbpD solved by molecular replacement is reported, which required *ab initio* models of each CbpD domain generated by the artificial intelligence deep-learning structure-prediction algorithm *RoseTTAFold*. The structure of CbpD confirms some previously reported substrate-specificity motifs among LPMOAA10s, while challenging the predictive power of others. Additionally, the structure of CbpD shows that post-translational modifications occur on the chitin-binding surface. Moreover, the structure raises interesting possibilities about how type 2 secretion-system substrates may interact with the secretion machinery and demonstrates the utility of new artificial intelligence protein structure-prediction algorithms in making challenging structural targets tractable.

## Abstract 2

The 3 Å resolution crystal structure of the *Pseudomonas aeruginosa* virulence factor CbpD both supports and challenges the current model of how lytic polysaccharide monooxygenases bind chitin and raises interesting possibilities about how type 2 secretion-system substrates may interact with the secretion machinery. This structure also demonstrates the utility of new, AI-powered, protein structure-prediction algorithms in making challenging structural targets tractable.

## 1. Introduction

*Pseudomonas aeruginosa* is a Gram-negative bacterium that is responsible for many nosocomial infections in the blood (McCarthy & Paterson, 2017 ▸), urinary tract (Vincent, 2003 ▸) and lungs. It remains one of the primary causes of death in cystic fibrosis patients (Lyczak *et al.*, 2000 ▸). A critical virulence factor in *P. aeruginosa* is its type 2 secretion system (T2SS; Jyot *et al.*, 2011 ▸), through which it exports diverse proteins. One T2SS substrate is the 39 kDa chitin-binding protein CbpD (Bleves *et al.*, 2010 ▸). CbpD was originally identified by its ability to bind only crystalline chitin (Folders *et al.*, 2000 ▸), and was thus classified into carbohydrate-binding module family 33 (CBM33; Cantarel *et al.*, 2009 ▸). CbpD is a virulence factor (Askarian *et al.*, 2021 ▸) that carries several different post-translational modifications (Ouidir *et al.*, 2014 ▸; Gaviard *et al.*, 2018 ▸, 2019 ▸) and whose function may be modulated by proteolytic cleavage after secretion through the T2SS (Park & Galloway, 1995 ▸; Braun *et al.*, 1998 ▸; Folders *et al.*, 2000 ▸).

A decade after the discovery of CbpD, it was shown that the members of the CBM33 family are mono-copper binding enzymes with oxidative activity on recalcitrant polysaccharide substrates (Vaaje-Kolstad *et al.*, 2010 ▸). These enzymes were reclassified as lytic polysaccharide monooxygenases (LPMOs; Horn *et al.*, 2012 ▸). Specifically, LPMOs catalyze the oxidation of glycosidic bonds in abundant natural polymers, including chitin (Hamre *et al.*, 2015 ▸), xylan (Corrêa *et al.*, 2019 ▸), cellulose (Forsberg *et al.*, 2011 ▸), starch (Vu, Beeson, Span *et al.*, 2014 ▸) and pectin (Sabbadin, Urresti *et al.*, 2021 ▸), with a range of substrate selectivity (Isaksen *et al.*, 2014 ▸; Agger *et al.*, 2014 ▸; Kojima *et al.*, 2016 ▸; Zhou *et al.*, 2019 ▸) and regioselectivity (Li *et al.*, 2012 ▸; Isaksen *et al.*, 2014 ▸; Vu, Beeson, Phillips *et al.*, 2014 ▸; Forsberg, Røhr *et al.*, 2014 ▸; Vaaje-Kolstad *et al.*, 2017 ▸). LPMOs utilize a reactive oxygen species to perform H-atom abstraction followed by hydroxylation of a carbon adjacent to the glycosidic bond (C1 in chitin and either C1 or C4 in cellulose) in the polysaccharide substrate (Bissaro *et al.*, 2020 ▸). Spontaneous elimination results in glycosidic bond cleavage (Beeson *et al.*, 2012 ▸), nicking the polysaccharide and creating new ends for processive degradation of the biopolymer (Agostoni *et al.*, 2017 ▸). While there has been some disagreement about the precise mechanism of LPMO oxidative cleavage (Bissaro *et al.*, 2017 ▸; Hangasky *et al.*, 2018 ▸), a thorough model has recently been proposed for the utilization of H_2_O_2_ as the reactive co-substrate (Bissaro *et al.*, 2020 ▸). LPMOs are further characterized by a solvent-exposed Peisach–Blumberg type 2 copper active site in which a mononuclear copper ion is coordinated in a T-shape by a histidine brace comprising the N-terminal amine and two histidines (Peisach & Blumberg, 1974 ▸; Hemsworth, Taylor *et al.*, 2013 ▸; Forsberg, Røhr *et al.*, 2014 ▸).

The discovery of catalytic activity in CBM33 precipitated a reorganization of the Carbohydrate Active enZYme (CAZy) database and the creation of a new enzyme class, the auxiliary activity (AA) enzymes, into which CBM33 members and other enzymes were sorted (Levasseur *et al.*, 2013 ▸). Based upon their sequence similarity, all CBM33 enzymes, including CbpD, were reclassified into the auxiliary activity 10 (AA10) family (Levasseur *et al.*, 2013 ▸). LPMOs have now been found in bacterial (Vaaje-Kolstad *et al.*, 2010 ▸), plant (Shukla *et al.*, 2016 ▸; Yadav *et al.*, 2019 ▸), fungal (Quinlan *et al.*, 2011 ▸; Beeson *et al.*, 2012 ▸), archaeal (Li *et al.*, 2022 ▸), insect (Sabbadin *et al.*, 2018 ▸) and viral (Chiu *et al.*, 2015 ▸) genomes and are classified into families AA9–AA11 and AA13–AA17 (Vandhana *et al.*, 2022 ▸).

**Figure 1:**
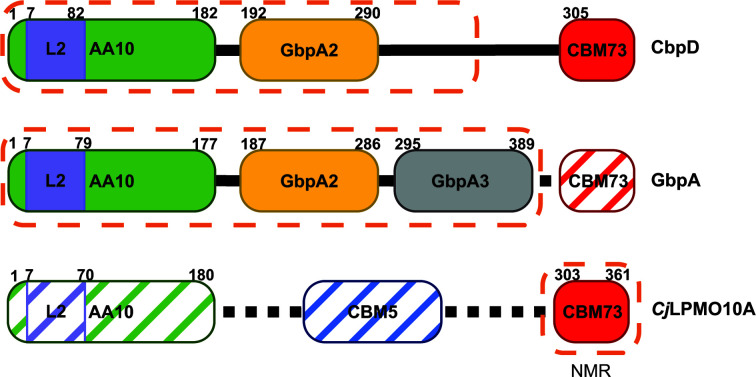
The domain architecture of CbpD and representative domain structures. CbpD is composed of three domains: an AA10 domain (green), a GbpA2 domain (yellow) and a CBM73 domain (red). The domain structures of the AA10 and GbpA2 families are represented in the crystal structure of *V. cholerae* GbpA (PDB entry 2xwx), while the domain structure of the CBM73 family is represented in the NMR solution structure of *Cj*LPMO10A (PDB entry 6z40). Hashes represent regions that are not included in the constructs used in the cited structural studies. Dashed orange lines encapsulate the domain(s) resolved in the respective reported structures. Residue boundaries for each domain are labeled based on previous annotations, adjusted so that the N-terminal His in each mature protein is residue 1.

The majority of LPMOs comprise a single copper-binding catalytic AA domain, while others have additional domains, themselves classified into families based on sequence identity (Horn *et al.*, 2012 ▸; Book *et al.*, 2014 ▸; Agostoni *et al.*, 2017 ▸; Vaaje-Kolstad *et al.*, 2017 ▸). These additional domains often contain aromatic residues that contribute to substrate binding and specificity (Cuskin *et al.*, 2012 ▸; Gilbert *et al.*, 2013 ▸; Crouch *et al.*, 2016 ▸) and are also likely to protect LPMOs from autocatalytic inactivation (Courtade *et al.*, 2018 ▸; Forsberg *et al.*, 2018 ▸). In addition to its AA10 domain, CbpD has a GbpA2 domain and a carbohydrate-binding module 73 (CBM73), as annotated in the PFAM (Mistry *et al.*, 2021 ▸) and CAZy (Drula *et al.*, 2022 ▸) databases, respectively (Fig. 1 ▸).

While the catalytic activity of LPMOs is driving techno­logical advances in biomass degradation (Harris *et al.*, 2010 ▸; Arfi *et al.*, 2014 ▸; Horn *et al.*, 2012 ▸; Rani Singhania *et al.*, 2021 ▸), their role in virulence has been less thoroughly investigated. LPMOs play a role in virulence (Wong *et al.*, 2012 ▸; Loose *et al.*, 2014 ▸; Chiu *et al.*, 2015 ▸; Agostoni *et al.*, 2017 ▸; Sabbadin, Henrissat *et al.*, 2021 ▸; Sabbadin, Urresti *et al.*, 2021 ▸) and are upregulated in pathogenic bacteria when exposed to human tissues (Vebø *et al.*, 2009 ▸, 2010 ▸). Similarly, evidence has existed for two decades that CbpD might play a role in *P. aeruginosa* virulence. The CbpD gene has extensively been found in pathogenic clinical isolates (Folders *et al.*, 2000 ▸) and is up­regulated in the lungs of cystic fibrosis patients (Sriramulu *et al.*, 2005 ▸). Recently, Askarian and coworkers demonstrated that catalytically active CbpD promotes the survival of *P. aeruginosa* in human whole blood, extends the survival of some other bacteria that do not endogenously express an LPMO, prevents cell lysis by the terminal complement pathway and is likely to aid the resistance of *P. aeruginosa* to immune clearing in mouse infection models (Askarian *et al.*, 2021 ▸).

These insights into the roles that LPMOs play in virulence have prompted studies of the structure and function of chitin-binding and virulence-associated LPMOs in pathogenic bacteria. The AA10 and GbpA2 domains of CbpD are represented structurally in the *Vibrio cholerae* colon­ization factor GbpA (PDB entry 2xwx; Wong *et al.*, 2012 ▸), while the CBM73 domain is found in a recently reported standalone NMR solution structure of the CBM73 domain of *Cellvibrio japonicus* *Cj*LPMO10A (PDB entry 6z40; Madland *et al.*, 2021 ▸). Despite the availability of these structures of representatives of the three domains of CbpD, no structure of this virulence factor has been reported.

Concurrently with progress in understanding the role of LPMOs in virulence, recent advances in artificial intelligence (AI)-powered *ab initio* protein structure prediction (Baek *et al.*, 2021 ▸) allowed us to solve the partial structure of the virulence factor CbpD. While structures of other LPMOs have been reported, many are of single-domain LPMOs or are of the catalytic domain of multidomain LPMOs (Vaaje-Kolstad, Houston *et al.*, 2005 ▸; Vaaje-Kolstad *et al.*, 2012 ▸; Aachmann *et al.*, 2012 ▸; Hemsworth, Taylor *et al.*, 2013 ▸; Gudmundsson *et al.*, 2014 ▸; Forsberg, Røhr *et al.*, 2014 ▸; Mekasha *et al.*, 2016 ▸). While the structure of *V. cholerae* GbpA is multimodular, it was generated from a truncated construct lacking the C-terminal domain (a CBM73; Wong *et al.*, 2012 ▸; Fig. 1 ▸). We report the AI-enabled crystal structure determination of a multidomain LPMO. This structure of CbpD provides insights into domain architecture, substrate-binding motifs, crystal packing, and apparent order and disorder propensities in multidomain LPMOs.

## 2. Methods and materials

### 2.1. Protein production and crystallization

CbpD from *P. aeruginosa* was previously cloned with its native signal peptide into the EcoRI site of pT7.5 with an encoded C-terminal 10×His tag to yield pCbpD (Cadoret *et al.*, 2014 ▸). Our purification procedure was modified only slightly from this publication. Overnight cultures of *Escherichia coli* BL21 (DE3) cells carrying pCbpD were used to inoculate 2 × 2 l cultures of Terrific Broth (Sigma–Aldrich), which were incubated at 37°C at 220 rev min^−1^ until the OD_600_ reached 0.5–0.6. Protein expression was induced with 1 m*M* isopropyl β-d-1-thiogalactopyranoside (IPTG) and proceeded overnight at 30°C. Cells were collected using low-speed centrifugation (6000*g*) and the pellet was resuspended in buffer *A* (50 m*M* Tris, 300 m*M* NaCl, 10 m*M* imidazole pH 8.2) and frozen at −80°C. After 2 h, the cells were thawed in a 37°C water bath for 20–30 min to induce lysis. DNAse, MgSO_4_ and lysozyme were added to final concentrations of 20 µg ml^−1^, 20 m*M* and 0.5 µg ml^−1^, respectively. The mixture was stirred at 4°C for 1 h to complete lysis. The clarified lysate obtained by centrifugation at 20 000*g* was loaded onto a 5 ml FFcrude prepacked nickel column (GE Healthcare) pre-equilibrated with buffer *A*. The column was washed with 10% buffer *B* (50 m*M* Tris, 300 m*M* NaCl, 250 m*M* imidazole pH 8.2), and CbpD was eluted using a gradient from 10 to 50% buffer *B* over 20 min. The elution fraction containing CbpD was concentrated to 5 ml using a 10K Amicon concentrator and loaded onto a Superdex S200 26/60 column equilibrated with 20 m*M* Tris, 100 m*M* NaCl pH 8. Fractions were analyzed by SDS–PAGE, which showed high purity of the purified protein (>95%), implying that no residual CbpD uncleaved by signal peptidase remained (Supplementary Fig. S1). Fractions containing CbpD were pooled, resulting in a yield of 2 mg total purified protein per litre of culture. For crystallization trials, the protein was concentrated to 8 mg ml^−1^.

Crystals were grown by the hanging-drop method against a reservoir solution consisting of 0.2 m*M* diammonium tartrate in 20%(*w*/*v*) PEG 3350 (condition H2 of the Qiagen PEGs Suite sparse-matrix screen). A crystal was harvested, mounted and flash-cooled in liquid nitrogen without additional cryoprotection.

### 2.2. Data collection and structure determination

An X-ray diffraction data set was collected to 2.98 Å resolution from a single crystal on the PROXIMA-1 beamline at the SOLEIL synchrotron. The data set was processed in *XSCALE* (Kabsch, 2010 ▸). Initial attempts to solve the structure by molecular replacement (MR) with *Phaser* (McCoy *et al.*, 2007 ▸) in *Phenix* (Liebschner *et al.*, 2019 ▸) using the first two domains of the *V. cholerae* multidomain LPMO GbpA individually (PDB entry 2xwx; Wong *et al.*, 2012 ▸) provided strong rotation-function solutions but no significant translation-function outputs. The amino-acid sequence of mature CbpD was then submitted to the *Robetta*, *Phyre* ^2^ (Kelley *et al.*, 2015 ▸) and *PSIPRED* (Buchan & Jones, 2019 ▸) servers and eight models were generated: five *ab initio* models from *RoseTTA­Fold* (Baek *et al.*, 2021 ▸), one homology model from *Phyre* ^2^ and two *ab initio* models from *DMPFold* (Greener *et al.*, 2019 ▸). MR was attempted using each CbpD model as a template. While no full-length model was able to generate a high-confidence solution, we chose the model with the highest LLG and TFZ scores (47 and 9, respectively) to break into domains. The AA10 domain of this *RoseTTAFold* model provided a partial solution (LLG, 181; TFZ, 18), which was held fixed in a subsequent round of MR using the *RoseTTA­Fold* GbpA2 output as the search model. This generated a successful MR solution (LLG, 432; TFZ, 22) containing the AA10 and GbpA2 domains of CbpD. An ensemble of CBM73 domains, including models generated by *RoseTTAFold*, *Raptor-X* (Askarian *et al.*, 2021 ▸) and the NMR solution structure of the *Cellvibrio japonicus* CBM73 domain (Madland *et al.*, 2021 ▸), was then used in subsequent rounds of MR, holding the AA10/GbpA2 partial solution fixed. Despite numerous attempts using varied input parameters, only low-likelihood solutions were found for the CBM73 domain and upon visual inspection were unlikely due to clashes with either the partial model of CbpD or symmetry mates.

The two-domain model of CbpD was rebuilt using *phenix.autobuild* (Liebschner *et al.*, 2019 ▸) to create the linker region between the AA10 and GbpA2 domains. The electron density was good enough to manually extend the model past the GbpA2 domain to residue 296 in *Coot* (Emsley *et al.*, 2010 ▸), which was used with *phenix.refine* (Liebschner *et al.*, 2019 ▸) for iterative rounds of real-space and reciprocal-space refinement. The refinements were performed with a high-resolution cutoff of 3 Å. Atomic displacement parameters were refined as group factors until the final round of refinement, when they were refined using one translation–libration–screw (TLS) group (Urzhumtsev *et al.*, 2013 ▸). The final model refined to *R*_cryst_ and *R*_free_ factors of 0.21 and 0.25, respectively (Table 1 ▸). Structure determination was performed using software supported by SBGrid (Morin *et al.*, 2013 ▸).

### 2.3. Modeling and docking

The orientation of CbpD on chitin could confidently be predicted because all chitin-active LPMOAA10s cleave at C1; thus, the reducing end of chitin must align with Glu171 and the nonreducing end must align with Tyr40/Glu20 (for an in-depth review of LPMO oxidation products, regioselectivity and substrate orientation, see Bissaro *et al.*, 2018 ▸).

Due to the lack of traceable electron density for the CBM73 domain, we evaluated the possible packing of this domain in the CbpD crystal lattice using the *RoseTTAFold* model of the CbpD CBM73 domain (Baek *et al.*, 2021 ▸). This model was first manually docked into the crystal voids using *Coot* (Emsley *et al.*, 2010 ▸) to minimize clashes with symmetry mates. A surface-model blob of each CBM73 domain was then generated in *PyMOL* (version 2.5; Schrödinger) by setting the *B* factor for all atoms to 100 Å^2^, setting the Gaussian resolution to 8.0, generating a Gaussian map for each CBM73 domain (grid = 1, buffer = 6) and finally generating an isosurface for each CBM73 domain (surface quality = 1).

To assess the fit of structural models to experimental small-angle scattering envelopes, a 15 Å volume map was generated in *ChimeraX* (Pettersen *et al.*, 2021 ▸) by converting the envelope bead model of CbpD (Askarian *et al.*, 2021 ▸; SASBDB entry SASDK42) into a density map using the *molmap* command. CbpD models were then manually placed into the density map and their positions were refined in the density map using the ‘Fit in Map’ tool in *ChimeraX*.

3D structure figures were generated in *PyMOL*. Structure-based topology was depicted through the use of *Pro-origami* (Stivala *et al.*, 2011 ▸).

## 3. Results

**Figure 2:**
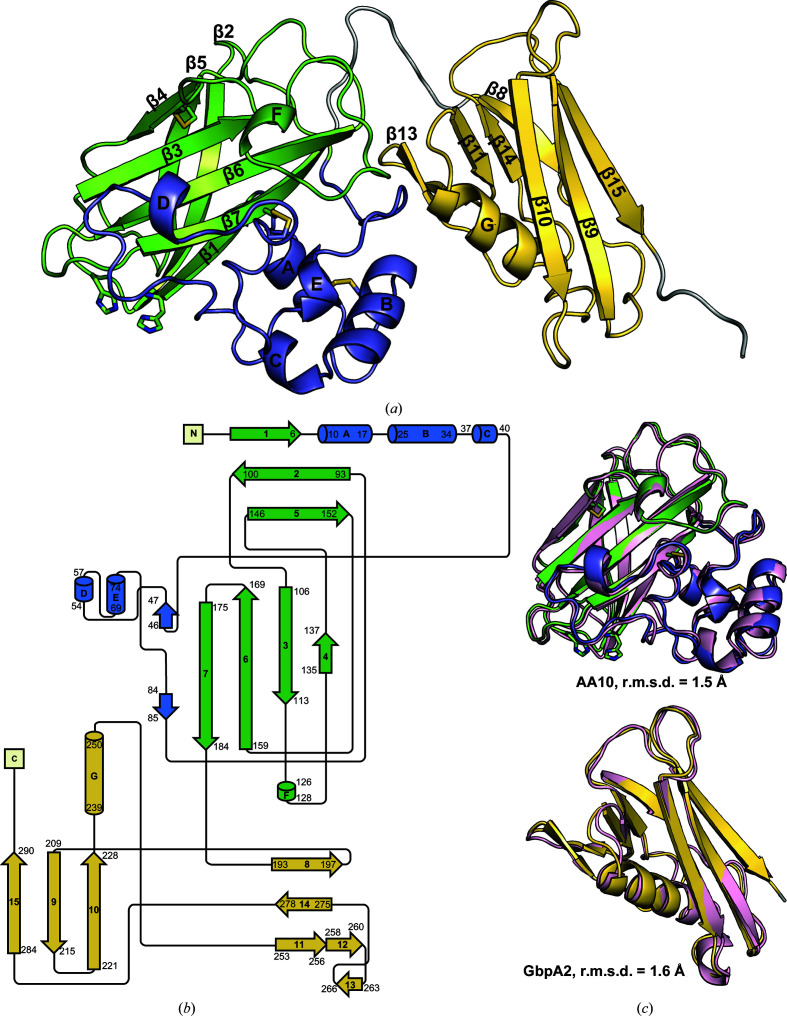
The overall structure of CbpD. (*a*) The structure of CbpD contains an AA10 domain (green) with an L2 region (slate) between β_1_ and β_2_ connected to a GbpA2 domain (yellow) by flexible linker regions (gray). Disulfide bridges and the active-site His residues are shown as sticks. (*b*) The structure-based topology diagram for CbpD was initially generated with *Pro-origami* (Stivala *et al.*, 2011 ▸), and the long intervening stretches were shortened manually and are not to scale. (*c*) A comparison of the AA10 and GbpA domains from the CbpD structure and predicted structural domain models from *RoseTTAFold* (Baek *et al.*, 2021 ▸; pink) reveals a high degree of predictive accuracy. The all-atom r.m.s.d.s between the predicted and crystallographic AA10 and GbpA2 domains are 1.5 and 1.6 Å, respectively.

Models generated from newly introduced protein structure-prediction AI algorithms enabled us to solve the structure of CbpD using molecular replacement, which had not been successful using the representative domain structures shown in Fig. 1 ▸. The structure of mature CbpD (residues 1–296, where 1 is the N-terminal His after signal peptide cleavage) was determined to 3.0 Å resolution with a single molecule in the asymmetric unit (Table 1 ▸). The overall structure contains the AA10 and GbpA2 domains, a well ordered random-coil linker between them, and similarly the portion of the linker between the GbpA2 and CBM73 domains to residue 296 (Figs. 2 ▸ *a* and 2 ▸ *b*). Residues 297–374 (the remainder of the linker and the CBM73 domain) were not visible in the electron-density map. We compared the AI-predicted structure with the final CbpD structure and found an all-atom r.m.s.d. of 11.3 Å (2215 atoms). We then compared each domain and found all-atom r.m.s.d.s of 1.5 Å (1370 atoms) and 1.6 Å (733 atoms) for the AA10 and GbpA2 domains, respectively (Fig. 2 ▸ *c*), compared with 8.9 Å (874 atoms) and 4.6 Å (569 atoms) for the refined CbpD domains and the representative domains in GbpA. In hindsight, it is noteworthy that a fern insecticidal LPMOAA10 (Yadav *et al.*, 2019 ▸; PDB entry 6if7) has a very low r.m.s.d. of 1.5 Å over 1252 atoms when compared with the CbpD AA10 domain, and is the most structurally similar to CbpD of those LPMOAA10s for which structures are now available (Supplementary Fig. S2). This structure was not yet available when our X-ray data were collected, but would have proved useful in phase determination by molecular replacement. MR with PDB entry 6if7 and the *RoseTTAFold* GbpA2 domain generated a successful MR solution containing the AA10 and GbpA2 domains of CbpD that had better statistics (LLG, 489; TFZ, 23) than MR with the *RoseTTAFold* domain predictions alone.

### 3.1. CbpD AA10 domain

The AA10 domain contains a typical LPMOAA10 fold, with a central seven-stranded β-sandwich comprising two antiparallel β-sheets connected by loop regions and an L2 subdomain between β_1_ and β_2_ (Figs. 2 ▸ *a* and 2 ▸ *b*). This L2 region forms the majority of the substrate-binding surface and displays a majority of the sequence variation in LPMOAA10s (Wu *et al.*, 2013 ▸; Forsberg *et al.*, 2016 ▸; Bissaro *et al.*, 2017 ▸; Vaaje-Kolstad *et al.*, 2017 ▸). The AA10 domain also contains three disulfide bonds: one linking β_4_ to β_5_ (Cys135 and Cys151), one linking two helices in the L2 domain (Cys14 and Cys27) and one linking the L2 domain to the β-sandwich through β_7_ (Cys64 and Cys179).

#### 3.1.1. Primary coordination sphere of the copper-binding active site

**Figure 3:**
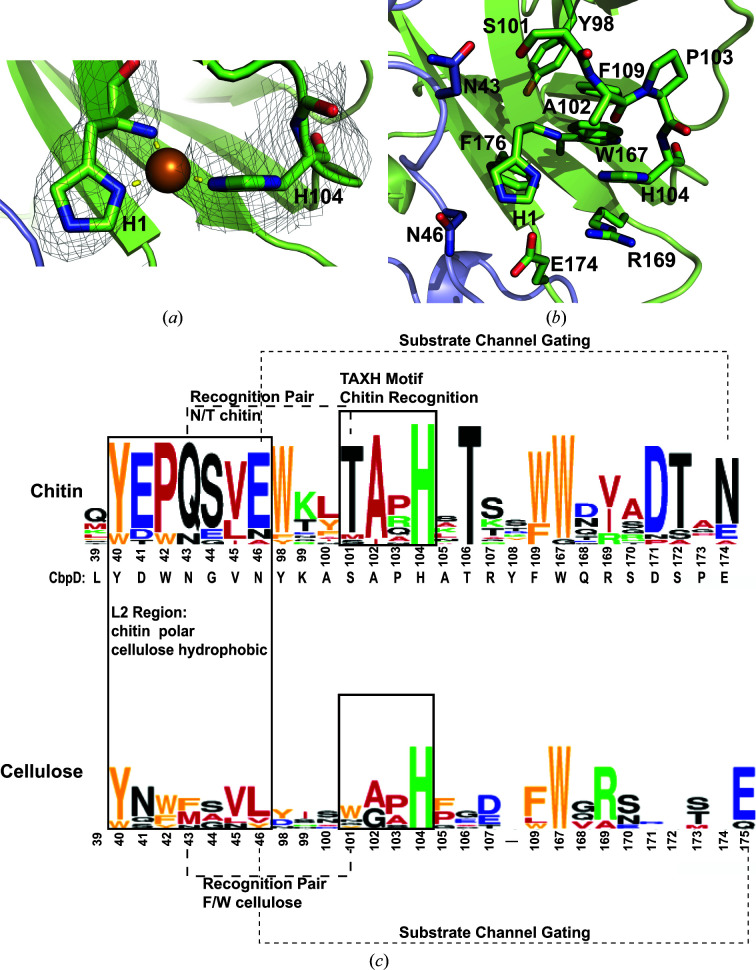
The structure of CbpD reveals that some chitin-specific motifs are preserved while others are more similar to cellulose-specific motifs. (*a*) The active site of CbpD contains a canonical His-brace motif. 2*F*_o_ − *F*_c_ electron density contoured at 0.7σ allows copper to be modeled into the active site (sphere_scale 0.6), approximately 2 Å away from the three coordinating N atoms, as expected. (*b*) The CbpD active site also contains a number of highly conserved residues. (*c*) Motifs previously identified as conserved among and/or proposed to confer substrate specificity in chitin- and cellulose-active LPMOAA10s are mapped to sequence logos generated from multiple sequence alignments of 13 chitin-active and seven cellulose-active LPMOAA10s listed as experimentally validated in the CAZy database (Drula *et al.*, 2022 ▸) and in Zhou & Zhu (2020 ▸), with the addition of CbpD to the chitin-active sequences. Motifs were concatenated for display and residues are numbered as in CbpD for the chitin-active sequence logo. In the cellulose-active sequence logo, residues are numbered by the structurally equivalent position in CbpD. The CbpD amino-acid identities are below the chitin logo. MSAs were generated with the *T-Coffee* web server (Notredame *et al.*, 2000 ▸; Di Tommaso *et al.*, 2011 ▸). Sequence logos were generated with *WebLogo* (Crooks *et al.*, 2004 ▸).

The active site comprises the canonical histidine brace found in LPMOs and is formed by His1 and His104 (Fig. 3 ▸ *a*). Although the active site did not appear to contain a coordinated metal, a copper could be modeled into the active site with the electron density contoured to 0.7σ (Fig. 3 ▸ *a*). Missing or partially occupied active-site copper is common in LPMOAA10 structures (Frandsen & Lo Leggio, 2016 ▸). In this position, the copper would only be coordinated axially in a T-shape by the histidine brace, as expected in an LPMOAA10 (Forsberg, Røhr *et al.*, 2014 ▸).

As in all LPMOAA10s, a secondary coordination sphere includes highly conserved features that help to structure the active site of CbpD (Fig. 3 ▸ *b*). These include a phenylalanine (Phe176; Hemsworth, Davies *et al.*, 2013 ▸) and an alanine (Ala102; Forsberg, Røhr *et al.*, 2014 ▸) in the two axial positions relative to the copper. These are believed to restrict the coordination of cosubstrates, with the alanine in particular blocking the solvent-exposed axial position, and thereby increase the likelihood that an oxygen cosubstrate coordinated at the equatorial position will be activated for catalysis, thus conferring C1/C4 oxidizing specificity (Forsberg, Mac­kenzie *et al.*, 2014 ▸; Borisova *et al.*, 2015 ▸). A well conserved tryptophan (Trp167) among LPMOAA10s (Forsberg, Røhr *et al.*, 2014 ▸; Meier *et al.*, 2018 ▸) is thought to protect the active site against oxidative inactivation during uncoupled turnovers (Loose *et al.*, 2018 ▸; Paradisi *et al.*, 2019 ▸; Gray & Winkler, 2021 ▸).

#### 3.1.2. Second shell of the copper-binding active site

In addition to the histidine brace and the characteristic LPMOAA10 residues described above, the CbpD active site contains three highly conserved features of chitin-specific LPMOAA10s. Firstly, in CbpD the reportedly invariant TA*X*H chitin-recognition motif (Bissaro *et al.*, 2018 ▸) is SAPH, with the conservative change of serine in place of threonine (A and H are the active-site Ala102 and histidine-brace His104, respectively; Figs. 3 ▸ *b* and 3 ▸ *c*). By interacting with Asn43 in the L2 loop, this serine secondly serves as one of the two amino acids in the ‘Gln–Thr pair’ previously identified as conserved among chitin-binding LPMOAA10s (Zhou & Zhu, 2020 ▸; Figs. 3 ▸ *b* and 3 ▸ *c*). In other words, the ‘Gln–Thr pair’ in CbpD becomes an Asn–Ser pair. It provides a polar staple from the L2 subdomain to the β-sandwich domain. In cellulose-active AA10s this pair is replaced by two large, hydrophobic residues, generally Phe–Trp (Forsberg *et al.*, 2016 ▸). The important role that the residues in the Gln–Thr pair and TA*X*H motifs play in substrate specificity have further been demonstrated by directed mutation experiments conferring chitinolytic activity to a cellulose-active LPMOAA10 (*Sc*LPMO10C; Jensen *et al.*, 2019 ▸). Thirdly, previous molecular modeling of a chitin-active LPMOAA10 bound to crystalline chitin identified a Glu–Asn pair (Glu60–Asn185 in *Sm*AA10A) that appears to gate a cosubstrate tunnel from the bulk solvent to the copper active site when an LPMOAA10 is bound to crystalline chitin (Fig. 3 ▸ *c*; Bissaro *et al.*, 2018 ▸). This gated tunnel is proposed to be the route through which only small-molecule cosubstrates (for example O_2_, 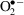 , H_2_O_2_ or H_2_O) could access the active site. All LPMOs have the Glu (sometimes Gln) which is required for enzymatic activity (Vaaje-Kolstad, Horn *et al.*, 2005 ▸; Harris *et al.*, 2010 ▸), likely by coordinating H_2_O_2_ and reactive oxygen species intermediates (Span & Marletta, 2015 ▸; Bacik *et al.*, 2017 ▸; Hedegård & Ryde, 2017 ▸; O’Dell *et al.*, 2017 ▸; Bissaro *et al.*, 2020 ▸). CbpD contains an analogous amino-acid pair Asn46–Glu174, but notably the two amino acids are swapped (Figs. 3 ▸ *b* and 3 ▸ *c*), a feature that is also present in *Cj*LPMO10A (Forsberg *et al.*, 2016 ▸). The swap retains the properties and position of the gating pair in the AA10 domain. Glu174 still points towards the active site of CbpD, approximately 5 Å away from the copper (Figs. 3 ▸ and 4 ▸ *a*). The maintenance of the polar interaction between the side chains and the proximity of Glu174 to the active site imply that Glu174/Asn46 may also gate the access of cosubstrates or electron donors to the active site when CbpD is bound to crystalline chitin. We also note that this amino-acid pair also serves to stabilize the L2/ β-sandwich interface. In these three chitin-recognition motifs, side-chain properties are conserved.

**Figure 4:**
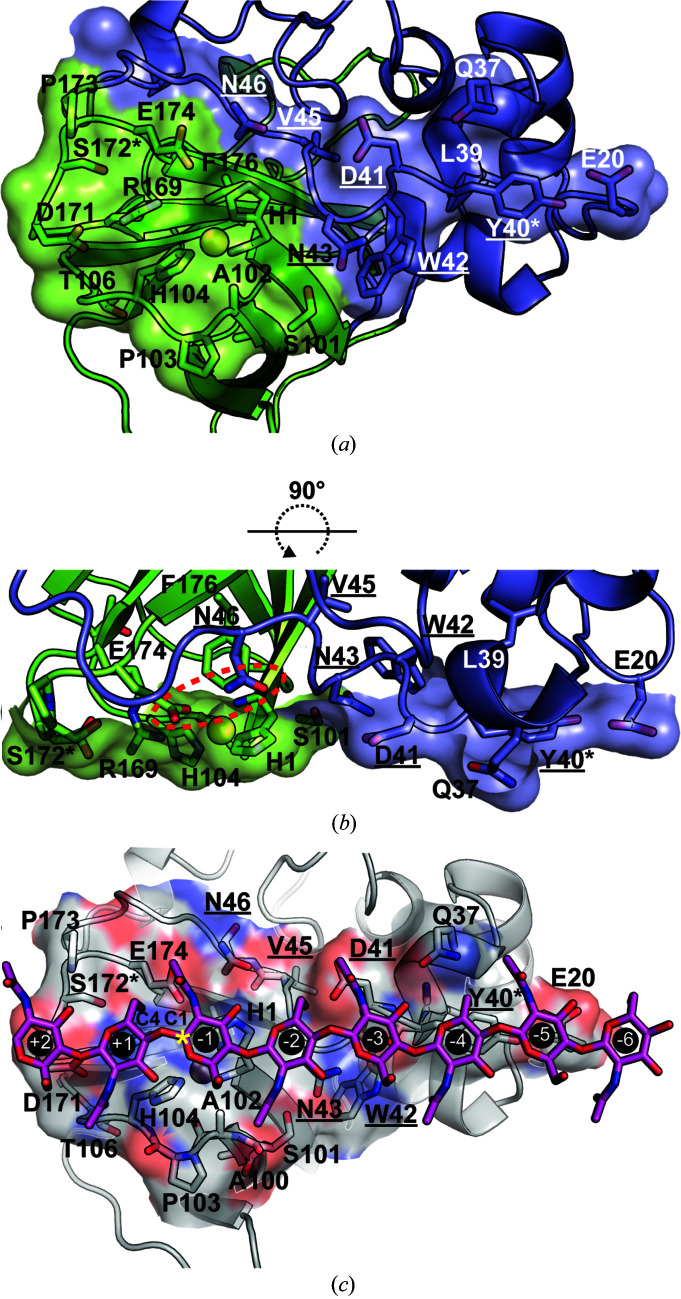
The chitin-binding surface of CbpD. (*a*) Looking at the chitin-binding surface from the perspective of the crystalline substrate, equivalent residues to those identified in *Sm*AA10 that interact with chitin (Vaaje-Kolstad, Houston *et al.*, 2005 ▸; Aachmann *et al.*, 2012 ▸; Bissaro *et al.*, 2018 ▸) are labeled. For reference and orientation, copper is modeled into the active-site His brace as a sphere (sphere_scale 0.6) and Phe176 is labeled and shown as sticks. Residues in the putative chitin-specific L2 motif are underlined. Ser172 and Tyr40, which are phosphorylated when CbpD is expressed and secreted by *P. aeruginosa* (Ouidir *et al.*, 2014 ▸), are marked with an asterisk. (*b*) Side view of the chitin-binding surface showing the relatively flat substrate-binding surface of the CbpD AA10 domain and the large role that the L2 region (slate) plays in substrate binding. The substrate tunnel gating pair Asn46/Glu174 are indicated with a dashed oval. (*c*) Based on the modeling of *Sm*AA10 interacting with β-chitin (Bissaro *et al.*, 2018 ▸), CbpD was manually docked onto crystalline anhydrous β-chitin (Nishiyama *et al.*, 2011 ▸; Crystallography Open Database entry 1501776) so that the substrate-binding surface of CbpD (O and N atoms of side chains in red and blue, respectively) was parallel to the chitin surface and aligned such that the chitin runs from one end of the surface (Glu171) across the active site to the opposite end (Tyr40/Glu20). A chito-octaose (NAG_8_) fragment interacting with subsites −6 to +2 is shown as sticks. Subsites are numbered following the standard practice for glycoside hydrolases (Sunna *et al.*, 1997 ▸).

CbpD, however, deviates from or lacks two features previously postulated to be conserved among chitin-active LPMOAA10s. CbpD lacks the cluster of three tryptophans previously identified as being conserved among bacterial chitin-active LPMOAA10s (Beeson *et al.*, 2015 ▸). Instead, CbpD has a trio of different aromatic amino acids: Tyr98, Phe109 and Trp167 (Fig. 3 ▸ *c*). This pattern of three aromatic residues has been identified in the strictly cellulose-active *Sc*LPMO10C (Forsberg, Røhr *et al.*, 2014 ▸). CbpD also contains a reversed putative discriminating feature between chitin and cellulose-active LPMOAA10s. Chitin-active LPMOAA10s were identified to contain a short, aliphatic residue (generally an isoleucine or valine) just below the histidine brace, while this position was an Arg in the cellulose-active LPMOAA10s *Tf*LPMO10B and *Sc*LPMO10C (Forsberg, Mackenzie *et al.*, 2014 ▸; Forsberg, Røhr *et al.*, 2014 ▸) and is preserved among the strict cellulose-active LPMOAA10s in the CAZy database (Drula *et al.*, 2022 ▸; Fig. 3 ▸ *c*). In chitin-active CbpD, however, this position is Arg169 (Fig. 3 ▸ *b*). It has been postulated that the short aliphatic residue in this position creates a cavity on the substrate-binding surface of chitin-active LPMOAA10s that could accommodate a dioxygen cosubstrate for catalysis (Hemsworth, Taylor *et al.*, 2013 ▸) or the *N*-acetyl group found in chitin (Forsberg, Mackenzie *et al.*, 2014 ▸). Arg169, Asp171 and Glu174 eliminate this cavity on the surface of CbpD (Fig. 4 ▸ *a*). This is also the case for chitin-active *Cj*LPMO10A (Forsberg *et al.*, 2016 ▸). In fact, in 16.7% of reviewed chitin-active LPMOAA10s in the CAZy database (Drula *et al.*, 2022 ▸) this position is an Arg (Supplementary Fig. S3). The Arg here as opposed to Ala or another short aliphatic residue implies that a surface cavity may not be required for catalytic activity or substrate discrimination.

#### 3.1.3. Chitin-binding surface

The structure of CbpD reveals a canonical relatively flat surface of ∼1000 Å^2^ encompassing the copper active site that is the presumed chitin-binding surface of CbpD (Figs. 4 ▸ *a* and 4 ▸ *b*). While no studies have directly examined the role that residues on this surface play in mediating chitin binding by CbpD, the structure of CbpD enables the mapping of homologous chitin-interacting residues from other characterized LPMOAA10s to CbpD. Residues that interact with chitin in chitin-specific LPMOAA10s have been identified through mutagenesis (Vaaje-Kolstad, Houston *et al.*, 2005 ▸), NMR studies (Aachmann *et al.*, 2012 ▸) and molecular-dynamics (MD) simulations (Bissaro *et al.*, 2018 ▸). The analogous putative chitin-binding residues of CbpD could be identified by mapping these sets of residues onto CbpD (Tyr40, Asp41 and His104 from all three methods, Asn46, Ser172 and Glu174 from mutagenesis and MD, Asn43, Gly44, Ser101 and Ala102 from NMR and MD, Leu39, Ala100 and Thr106 from NMR, and Pro173 from MD; Figs. 4 ▸ *a* and 4 ▸ *b*). These residues form a surface that extends from the conserved single aromatic residue (Tyr40) found on the substrate-binding surface of chitin-active LPMOAA10s (Zhou & Zhu, 2020 ▸) to Asp171/Ser172 at the edge of the substrate-binding surface. The structure suggests that Glu20 may further extend the substrate surface beyond Tyr40 in CbpD.

Interestingly, as for the sequence motifs examined above, mapping the features of chitin-specific LPMOAA10 substrate surfaces to CbpD revealed deviations in CbpD from previously identified trends. A motif on the L2 domain, beginning with the conserved substrate-binding Tyr and running down the center of the substrate-binding surface, purportedly exhibits differing polar characteristics in chitin- and cellulose-specific LPMOAA10s (Zhou *et al.*, 2019 ▸). In the chitin-specific LPMOAA10s examined this motif contained at least 70% polar residues, with the consensus sequence Y(W)EPQSVE (Fig. 3 ▸ *c*). The cellulose-specific LPMOAA10s examined had a motif containing more than 70% hydrophobic residues. In CbpD, this L2 motif is YDWNGVN, beginning with the conserved Tyr40. This sequence differs from the consensus chitin-specific sequence, and the side-chain properties fall between the properties of the chitin- and cellulose-specific motifs (Fig. 3 ▸ *c*). This motif in CbpD still forms a flat surface that is an appropriate area for substrate binding (Fig. 4 ▸ *b*), and the side-chain characteristics of position 2 in the consensus sequence are conserved. This residue may be particularly important for chitin recognition and enzymatic activity because mutating this residue significantly reduces chitin binding (Vaaje-Kolstad, Horn *et al.*, 2005 ▸) and eliminates enzymatic activity (Loose *et al.*, 2018 ▸) in another chitin-active LPMOAA10 (*Sm*LPMO10A). The divergence of most L2 motif residues in CbpD from previous trends raises questions about the extent to which the entire motif in LPMOAA10s determines substrate specificity. Interestingly, the chitin-active insecticidal putative LPMOAA10 produced by the fern *Tectaria macrodonta* (Tma12) has the sequence YEWNEVN that closely matches the outlier CbpD sequence here, providing further evidence that the Y(W)EPQSVE motif is not the determinant of chitin specificity.

### 3.2. CbpD GbpA2 domain

**Figure 5:**
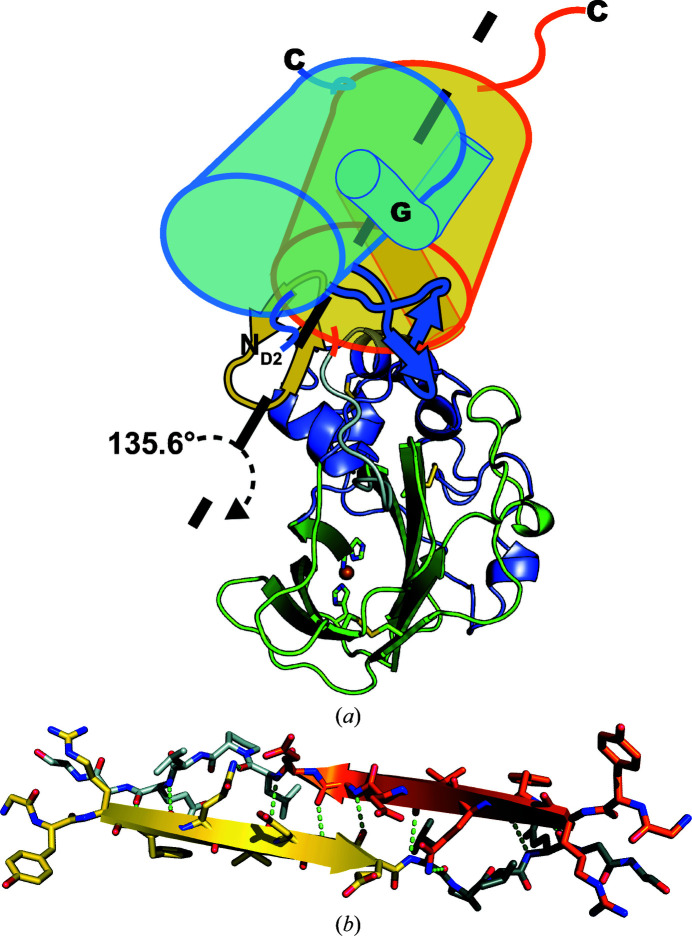
The role of the GbpA2 domain in crystal packing. (*a*) The GbpA2 domain in the CbpD structure (yellow cylinder) is displaced by 1 Å and rotated by 135.6° relative to the GbpA2 domain in the GbpA structure (light blue cylinder) when their AA10 domains are aligned. The αG of each GbpA2 domain (smaller cylinders), the β_12_–β_13_ hairpins (stylized cartoons) and the C-termini are indicated for orientation. The AA10 domain of CbpD is represented as in Fig. 2 ▸(*a*), with copper modeled into the active site as a sphere (sphere_scale 0.6). The displacement of GbpA2 domains is also noted by the N_D2_ label at the shifted N-terminus of this domain in GbpA. (*b*) β_15_ and part of the linker between the GbpA2 and CBM73 domains of one CbpD (yellow and dark gray) engage in extensive β-strand interactions with a symmetry mate (orange and light gray) to form an extended β-sheet, burying 990 Å^2^ of surface area per monomer.

The second domain of CbpD is, as expected, similar to the GbpA2 domain structure in GbpA (Wong *et al.*, 2012 ▸), with an all-atom r.m.s.d. of 4.6 Å for 569 atoms (residues 193–292 in CbpD and 211–314 in GbpA). The GbpA2 domain comprises an α-helix partially sandwiched between two antiparallel β-sheets (β-strands β_9_, β_10_ and β_15_ and β-strands β_8_, β_11_ and β_14_), with an unusual bend that allows β_11_ to pair with β_14_ while its immediate extension β_12_ interacts with the short β_13_ to form a hairpin above the helix (Figs. 2 ▸ *a* and 2 ▸ *b*). The orientation of the GbpA2 domain relative to the AA10 domain is shifted in the structure of CbpD by 1 Å and 135.6° compared with the structure of GbpA (Fig. 5 ▸ *a*). This shift means the CbpD crystal structure is closer to the elongated conformation observed for both GbpA and CbpD by solution scattering (Wong *et al.*, 2012 ▸; Askarian *et al.*, 2021 ▸; Supplementary Fig. S4). In contrast, GbpA adopts a U-shaped conformation in its crystal, forming an extensive interface in which each subunit contributes 3120 Å^2^ to the dimerization interface, as calculated with *PISA* (Krissinel & Henrick, 2007 ▸). Notably, while CbpD does not form a similar dimer, it does have a crystal contact formed by continuation of the β-sheet from β_15_ of one CbpD GbpA2 domain into its symmetry mate, burying 990 Å^2^ per monomer (Fig. 5 ▸ *b*).

### 3.3. CbpD CBM73 domain

**Figure 6:**
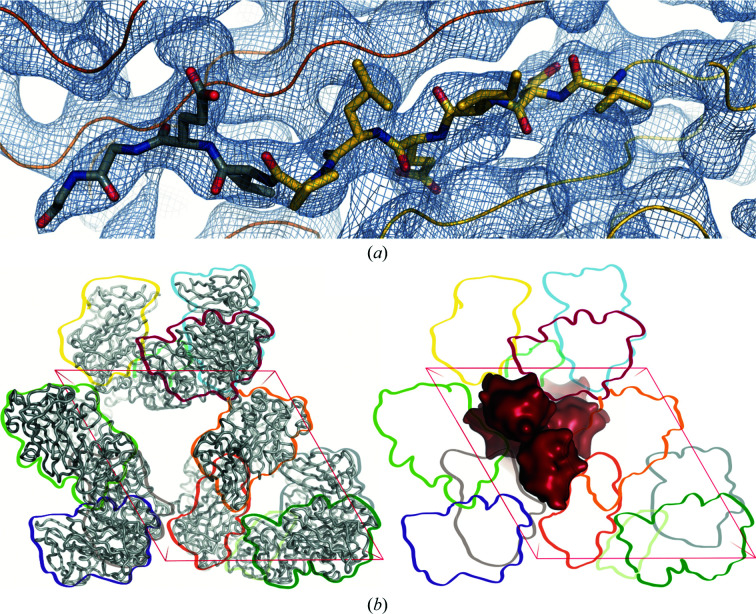
The CBM73 domain is present but unresolved in the CbpD crystal structure. (*a*) The 2*F*_o_ − *F*_c_ electron-density map is contoured at 1.0σ. The C_α_ trace of the CbpD GbpA2 domain is shown (yellow and gray with residues 287–296 as sticks) with a symmetry mate (orange). The electron density extends to residue 296. (*b*) Despite the absence of strong density to place the CBM73 domain, there is space in the crystal packing where the domain fits. Looking roughly down the *c* axis of the unit cell, the left panel depicts one unit cell of the modeled residues of CbpD (ribbons), with each symmetry mate outlined for clarity and reference in the right panel (arbitrary colors). The right panel depicts how the CBM73 domain (red blobs) could pack into the remaining crystal volume.

While full-length CbpD-His_10_ was crystallized, good electron density was only observed to residue 296 (Fig. 6 ▸ *a*), leaving nine residues of the linker between the GbpA2 and CBM73 domains, as well as the CBM73 domain itself, un­modeled. Despite the absence of strong density to place the CBM73 domain, it is unlikely that the residues were missing from the protein crystal. Given that the His_10_ affinity tag was attached to the C-terminus of CbpD, it is clear that full-length CbpD was purified via metal-affinity chromatography (see Section 2 and Supplementary Fig. S1). Crystal solvent-content analysis with *phenix.xtriage* (Zwart *et al.*, 2005 ▸) calculated 46% solvent content for a unit cell containing 374 residues, consistent with the crystallization of full-length CbpD-His_10_. While there is some evidence that CbpD is processed from its C-terminus by LasB into the 23 kDa LasD when secreted by *P. aeruginosa* (Braun *et al.*, 1998 ▸), CbpD-His_10_ was expressed in *E. coli*, which lacks LasB. The processing of CbpD into LasD would, moreover, result in the loss of all but the first 24 residues of the GbpA2 domain, which is fully present in the crystal structure. Additionally, there is a sufficiently large volume remaining in the crystal packing of CbpD to arrange the CBM73 domain (Fig. 6 ▸ *b*), although in a more compact configuration than the fully elongated configuration deduced from SAXS data (Askarian *et al.*, 2021 ▸).

## 4. Discussion

The structure of CbpD reported here is the first structurally determined LPMO from *P. aeruginosa* and highlights the utility that new, AI-powered, *ab initio* protein structure-prediction algorithms have in the structural biology toolkit. As demonstrated with CbpD, these *ab initio* models may help to solve previously recalcitrant X-ray crystallographic structures through MR by providing starting models that are substantially closer to the actual protein structure than homologous structures. Because these AI-generated models may provide better MR models, they may also enable protein structures to be solved with lower resolution or lower quality data than was previously routine. Additionally, these models will provide another path towards phasing protein structure targets without the need for additional experimental steps such as selenomethionine labeling, the collection of multiple data sets or heavy-metal soaking (which is also a danger to human and environmental health). These AI-generated models could be particularly beneficial for crystal targets that are difficult to express and purify in large quantities, produce few high-quality diffracting crystals or lack closely homologous structures that otherwise could serve as the basis for MR. The close agreement between AI-generated *ab initio* models and the crystal structures of each domain of CbpD, specifically the AA10 catalytic domain, also indicates that these AI results may provide high-quality structural models for the thousands of LPMOs that have been identified but lack experimental structures to develop more robust hypotheses about the characteristics of LPMOs that affect catalysis and substrate selectivity.

The structure of CbpD provides greater insight into the conserved features of chitin-active LPMOAA10s. CbpD displays a number of motifs (Glu–Thr, TA*X*H and the substrate tunnel gating pair) that demonstrate conservation of the structural properties of chitin-active LPMOAA10s despite deviations in motif sequences (Fig. 3 ▸ *c*). The similarity of Tyr40, Asp41, Asn43 and Ser101 in the Gln–Thr pair and TA*X*H motifs to the chitinolytic sequence identified by Jensen *et al.* (2019 ▸) indicates that these motifs near the active site of CbpD are likely to contribute to its chitin specificity. The structure of CbpD reinforces the spatially conserved nature of the Glu residue in the active site that is oriented towards the His brace irrespective of its occurrence in the amino-acid sequence, in line with observations for other LPMOAA10s (O’Dell *et al.*, 2017 ▸; Forsberg *et al.*, 2019 ▸). While Glu174 and Asn46 are flipped compared with the Glu60/Asn185 pair in *Sm*AA10A, the preservation of the structural position of the pair in the AA10 domain indicates that Glu174/Asn46 may play a similar role in gating the access of small-molecule cosubstrates such as O_2_ or H_2_O_2_ to the active site when CbpD is bound to crystalline chitin as Glu60/Asn185 in *Sm*AA10A (Bissaro *et al.*, 2018 ▸). Notably, a previous study found that mutating Glu60 to Asn was deleterious to catalytic activity (Bissaro *et al.*, 2020 ▸). This study, however, did not examine the effect of a concomitant mutation to Glu of the other residue in the Glu/Asn pair, which likely explains why CbpD retains chitinolytic activity despite having an apparently deleterious Asn residue at position 46 (equivalent to Glu60) and highlights the need for careful spatial as well as sequence considerations when conducting enzymatic mutation studies.

Other features, however, deviate substantially from previously characterized LPMOAA10s (Fig. 3 ▸ *c*). In the proximity of the active site, the presence of Arg169 and the absence of a pocket on the substrate surface is noteworthy because previously an Arg in this position was associated with only cellulose activity in LPMOAA10s (Forsberg, Røhr *et al.*, 2014 ▸). Interestingly, an Arg is present in this position in two other chitin-active LPMOAA10s (Tma12 and *Cj*LPMO10A; Supplementary Fig. S3), and all three are structurally similar to the cellulose-active LPMOAA10 *Sc*LPMO10C based on a *DALI* server ‘all against all’ comparison (Holm, 2020 ▸) and are the only members of their structural class to lack cellulolytic activity (Supplementary Fig. S2). This raises the possibility that there are distinct yet convergent features of LPMOAA10s that have evolved to confer chitin-specific activity and indicates that these features may not all have been identified. Interestingly, CbpD may have a shallow pocket just to the side of the active site that may be able to accommodate an *N*-acetyl group (Fig. 4 ▸ *c*), although it is between the putative channel gating resides (Glu174/Asn46) and the copper active site and may be part of the substrate tunnel when CbpD is bound to chitin. The structures of CbpD, Tma12 and *Cj*LPMP10A now provide three examples of LPMOAA10s that are strictly active on chitin, yet lack the pocket previously thought to facilitate either substrate hydrolysis by accommodating the oxygen cosubstrate or substrate binding by accommodating the *N*-acetyl group present in chitin, and should inform future investigations into the deterministic features that confer substrate specificity in LPMOAA10s.

The cluster of aromatic residues Tyr98, Phe109 and Trp167 also deviate from the previously identified three conserved Trp residues in chitin-active LPMOAA10s (Beeson *et al.*, 2015 ▸) and instead match the pattern found in cellulose-active *Sm*LPMO10C (Forsberg, Røhr *et al.*, 2014 ▸). This, combined with their location away from the putative substrate-binding surface, indicates that the Trp trio is likely not to be a determinant in substrate specificity and may rather play a role in stabilizing the hydrophobic core of the domain. These aromatic residues near the active site may also be important in protecting CbpD from oxidative damage (Paradisi *et al.*, 2019 ▸). It remains to be seen whether this protective mechanism is the reason that aromatic residues appear to be conserved in general, if not with strict identity, near the active site of LPMOs.

A Trp trio in LPMOs was previously suggested to be important for a hypothesized electron-transfer (ET) catalytic mechanism based on O_2_ as the oxygen cosubstrate (Vaaje-Kolstad *et al.*, 2012 ▸; Forsberg, Røhr *et al.*, 2014 ▸). This ET path was putatively identified in fungal LPMOs (Li *et al.*, 2012 ▸; Beeson *et al.*, 2012 ▸; Book *et al.*, 2014 ▸; Lo Leggio *et al.*, 2015 ▸; Frandsen *et al.*, 2016 ▸), but evidence has since been presented that cellobiose dehydrogenase (CDH) actually binds fungal LPMOAA9 active sites directly as an external electron donor, abrogating the need for an ET path in LPMOAA9s (Courtade *et al.*, 2016 ▸; Kracher *et al.*, 2016 ▸). An ET mechanism is also unlikely in bacterial LPMOAA10s, including CbpD. An equivalent ET pathway was not identified in bacterial chitin-active LPMOs (Vaaje-Kolstad *et al.*, 2012 ▸), although the concept has been revisited in recent literature based on an LMPOAA10 with two copper sites (Fowler *et al.*, 2019 ▸). The requirement that electrons would traverse a conserved Phe (Phe176), which does not facilitate ET (Beeson *et al.*, 2015 ▸), and the presence of gating residues similar to *Sm*AA10A (Bissaro *et al.*, 2018 ▸) indicates that there is a path into the active site of CbpD that is accessible to small-molecule reductants, eliminating the questions about how electrons could reach the copper active site for O_2_-based catalysis when bound to a crystalline substrate that originally led to the hypothesis of the ET mechanism (Li *et al.*, 2012 ▸; Walton & Davies, 2016 ▸). The lack of an identified bacterial CDH (Beeson *et al.*, 2015 ▸) and the identification of a number of alternative electron-donor sources, including small molecules (Westereng *et al.*, 2015 ▸, 2016 ▸; Askarian *et al.*, 2021 ▸), photo-active pigments (Cannella *et al.*, 2016 ▸) and even substrates themselves (Westereng *et al.*, 2015 ▸), rule out that bacterial LPMOAA10s rely on an internal ET path if they utilize O_2_ as a cosubstrate.

What remains unclear, however, is whether LPMOs may have reducing-agent specificity in addition to substrate specificity (Frommhagen *et al.*, 2016 ▸; Meier *et al.*, 2018 ▸). Askarian and coworkers demonstrated that CbpD chitinolytic activity was enhanced by ascorbate and the redox-active compound pyocyanin, which is secreted by *P. aeruginosa* (Askarian *et al.*, 2021 ▸), and Branch and coworkers demonstrated that a *c*-type cytochrome can activate the cellulose-active *Cj*AA10B in *Cellvibrio japonicus* (Branch *et al.*, 2021 ▸). CbpD and other LPMOs may favor reductants that are secreted by their hosts.

Future mechanistic studies can use the structure of CbpD to investigate whether the proposed mechanisms are universal and could occur in structurally diverse LPMOs with the same and divergent substrates, or whether the mechanisms may be family dependent, and whether LPMOs utilize general or context-specific reductants if an H_2_O_2_-based mechanism is employed.

The variation of the chitin-specific L2 motif Y(W)EPQSVE on the substrate-binding surface of CbpD further challenges previously strong conclusions about the deterministic features of LPMOAA10s that confer substrate selectivity. It is unclear whether the L2 motif is more tolerant of sequence variation than first supposed (Zhou *et al.*, 2019 ▸) or whether the L2 motif plays a critical role in substrate specificity among LPMOAA10s. These discrepancies in the active-site secondary coordination sphere and substrate-binding surface also raise an additional question about whether CbpD may be able to bind and hydrolyze substrates beyond chitin.

Many previous structural studies (Forsberg, Mackenzie *et al.*, 2014 ▸; Forsberg, Røhr *et al.*, 2014 ▸; Zhou *et al.*, 2019 ▸; Zhou & Zhu, 2020 ▸) drew their conclusions about discriminatory features from a relatively small number (tens) of evolutionarily closely related LPMO sequences. The structure of CbpD highlights the importance of including an evolutionarily diverse array of LPMOs in the identification and analyses of substrate-specificity motifs. This is particularly true given that the bacterial LPMOAA10 CbpD is structurally most similar to the fern LPMOAA10 Tma12 (Supplementary Fig. S2). The presence of the same L2 motif sequence from CbpD in the structurally similar yet evolutionarily distant fern LPMOAA10 Tma12 demonstrates that the structural features of LPMOAA10s that enable chitin binding and degradation have yet to be fully understood and require further investigation among structurally diverse members of the AA10 family.

Even with the structures of CbpD, Tma12 and *Cj*LPMO10A challenging previously identified substrate-specificity determinants, there are still relatively few LPMOAA10s that have experimentally verified substrate specificity. To date, the CAZy database contains over 8000 LPMOAA10 proteins, yet only 32 are listed as ‘characterized’ and this list does not include CbpD, despite its substrate binding being characterized in 2000 (Folders *et al.*, 2000 ▸) and its catalytic activity profile being confirmed in 2021 (Askarian *et al.*, 2021 ▸). One characterized LPMOAA10, *Kp*LPMO10A, appears to be able to cleave chitin, cellulose and xylan (Corrêa *et al.*, 2019 ▸), expanding the substrate scope of LPMOAA10s beyond just chitin and cellulose, yet no studies have investigated the structural features that may enable this broader substrate scope in an LPMOAA10. Until a larger portion of the LPMOAA10 family has characterized substrate profiles, sequence or structural motifs and features identified to confer absolute substrate specificity must be considered provisional.

Because the catalytic activity of CbpD is necessary for *P. aeruginosa* virulence (Askarian *et al.*, 2021 ▸), discrepancies in putative substrate-specificity features highlight the importance of considering the role of LPMOs in both carbohydrate polymer degradation and virulence when identifying the residues and motifs that confer substrate specificity. Humans do not make the chitin polymer, but do produce highly glycosylated mucin proteins (Corfield, 2015 ▸). Mucins play a critical role in cystic fibrosis (Morrison *et al.*, 2019 ▸), and CbpD may facilitate *P. aeruginosa* infection in these patients. Moreover, *V. cholerae* GbpA, which shares an LPMOAA10 domain and the GbpA2 domain with CbpD, is a demonstrated mucin-binding protein (Bhowmick *et al.*, 2008 ▸) which relies on its LPMOAA10 domain for mucin binding (Wong *et al.*, 2012 ▸). While mucin glycans are comprised of diverse saccharides and glycosidic bonds, they do contain β1–4 linkages involving *N*-acetyllactosamine and may imperfectly mimic chitin. Askarian and coworkers found that CbpD does not bind individual glycan structures within mucin glycans, and CbpD may only be able to imperfectly bind full mucin glycans. An alternative biological role for CbpD might be to enable bacteria–fungi interactions in the polymicrobial biofilms and co-infections that *P. aeruginosa* forms with pathogenic fungi, including *Aspergillus fumigatus* (Zhao & Yu, 2018 ▸) and *Candida albicans* (Harriott & Noverr, 2011 ▸), as fungal cell walls are chitinous.

The presence of a few discrepancies in previously identified substrate-specificity motifs coupled with its role in virulence mean that CbpD should serve as a basis for more extensive modeling of the interaction of LPMOAA10s with chitin. LPMOAA10–chitin modeling to date has only been performed with single-domain LPMOAA10s (Bissaro *et al.*, 2018 ▸). Additionally, this modeling was performed without the consideration of post-translational modifications (PTMs). The structure of CbpD, however, reveals that two residues that are phosphorylated when CbpD is secreted from *P. aeruginosa* (Ouidir *et al.*, 2014 ▸), Ser172 and Tyr40, form part of the chitin-binding surface of CbpD (Fig. 4 ▸), and in the case of Tyr40 are critical for substrate binding (Beeson *et al.*, 2015 ▸; Bissaro *et al.*, 2018 ▸). It has been speculated that phosphorylation at Ser172 proximal to Glu174 in the secondary coordination sphere of the copper active site is likely to affect cosubstrate (O_2_ or H_2_O_2_) activation and may alter the copper redox potential (Askarian *et al.*, 2021 ▸). A number of these post-transitionally modified residues are also found in the AA10–GbpA2 domain interface of CbpD, likely encouraging a more elongated overall conformation, similar to the observed solution scattering conformation. Additionally, a recent bioinformatics analysis of 27 000 LPMOs found that most of the eight LPMO AA families are enriched in Ser and Thr residues in C-terminal regions that are prime candidates for PTM, specifically *O*-glycosylation (Tamburrini *et al.*, 2021 ▸), which may affect secretion (Vorkapic *et al.*, 2019 ▸). Of note, the Tma12 structure has an *N*-glycosylation at Asn158, structurally equivalent to Ser136 in CbpD, which has been identified as phosphorylated when CbpD is expressed and secreted by *P. aeruginosa* (Ouidir *et al.*, 2014 ▸). A complete understanding of how LMPOAA10s are secreted by their native organisms and bind to their substrates cannot be obtained without taking these PTMs into consideration.

The β-strand interaction observed between the GbpA2 domains of crystallographic symmetry mates raises another question about the possible biological function of structural features of CbpD. Edge β-strands are well known to mediate protein–protein interactions, which in some cases are dynamic and interchangeable (Richardson & Richardson, 2002 ▸; Monteiro *et al.*, 2013 ▸; Yu *et al.*, 2014 ▸; Miyazaki *et al.*, 2021 ▸). Given the broad substrate repertoire of the *P. aeruginosa* T2SS (Cianciotto & White, 2017 ▸) and the expectation that the recognition of secreted proteins by multiple proteins along the secretion-machinery pathway relies on dynamic, short-lived interactions (Douzi *et al.*, 2011 ▸; Michel-Souzy *et al.*, 2018 ▸) as well as inherent disorder (Gu *et al.*, 2017 ▸; Pineau *et al.*, 2021 ▸), it is interesting to imagine that the β_15_ edge strand of CbpD may serve as a handle for interaction with secretion-machinery proteins along the pathway through the cell envelope. The CbpD structure may thus provide an experimental entrée into a better understanding of how T2SS substrates are recognized.

## 5. Conclusions

In addition to demonstrating the utility of new structural biology tools, the structure of CbpD provides evidence to both support and challenge aspects of the current model of how LPMOAA10s bind their substrates, specifically those that are preferential for chitin. The absence of the CBM73 domain in the structure also raises questions about the inherent stability of smaller carbohydrate-binding domains, and a crystal structure of a CBM73 domain remains to be solved. One possibility is to perform high-resolution, single-particle electron microscopy of CbpD bound to chitin fibrils. This could provide the structure of full-length CbpD and shed light on the conformation of CbpD when bound to crystalline chitin. A better understanding of the structure, conformations and dynamics of multimodular LPMOs could lead to advances in understanding the role of these enzymes in biomass degradation and virulence.

## Supplementary Material

PDB reference: CbpD, 7sqx

Supplementary Figures. DOI: 10.1107/S2059798322007033/jc5051sup1.pdf

## Figures and Tables

**Table 1:** Data-collection, processing and refinement statistics Values in parentheses are for the highest resolution bin.

Data-collection statistics	
Diffraction source	PROXIMA-1, SOLEIL
Wavelength (Å)	1.0087
Temperature (K)	100
Detector	Dectris PILATUS 6M
Space group	*P*3_1_12
*a*, *b*, *c* (Å)	85.77, 85.77, 90.86
α, β, γ (°)	90, 90, 120
Resolution range (Å)	42.89–2.90 (3.00–2.90)
Total No. of reflections	97198
No. of unique reflections	8654 (855)
Completeness (%)	99.9 (99.9)
Multiplicity	11.2
〈*I*/σ(*I*)〉	11.9
Wilson *B* factor (Å^2^)	78.9
Matthews coefficient (Å^3^ Da^−1^)	0.47
Refinement statistics	
Resolution range (Å)	38.78–3.00 (3.19–3.00)
Completeness (%)	99.9
No. of reflections, working set	7041 (1157)
No. of reflections, test set	781 (130)
Final *R*_work_/*R*_free_	0.21/0.25 (0.35/0.40)
No. of atoms	
Protein	2215
Ligand	15
Waters	5
R.m.s.d., bond lengths (Å)	0.003
R.m.s.d., angles (°)	0.57
Average *B* factor (Å^2^)	66.0
Ramachandran plot (%)	
Most favored	93.5
Allowed	6.5
Outliers	0
