# Supplementary material for: The crystal structure of CbpD clarifies substrate-specificity motifs in chitin-active lytic polysaccharide monooxygenases
Source: Acta Crystallogr D Struct Biol. 2022 Jul 27;78(Pt 8):1064–78. doi: 10.1107/S2059798322007033 (PMC9344471; doi:10.1107/S2059798322007033)
Supplement: Supplementary file 1 [file d-78-01064-sup1.pdf]

STRUCTURAL BIOLOGY

Volume 78 (2022)

Supporting information for article:

The crystal structure of CbpD clarifies substrate-specificity motifs in chitin-active lytic polysaccharide monooxygenases

Christopher M. Dade, Badreddine Douzi, Cristian Cambillau, Genevieve Ball, Romé Voulhoux and Katrina Forest

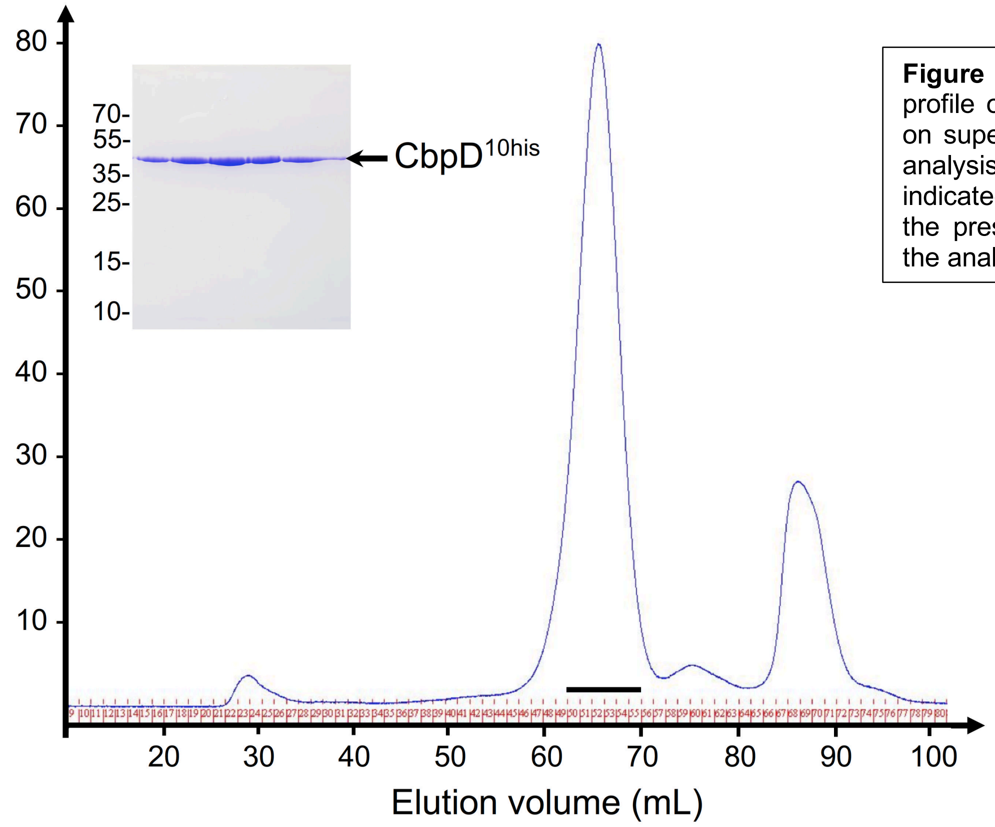

Figure S1 CbpD purification: SEC profile of CbpDHis purified by IMAC on superose 200 16/60. SDS-PAGE analysis of the SEC fractions indicated by the black line revealed the presence of full-length CbpD in the analyzed peak (43kDa).

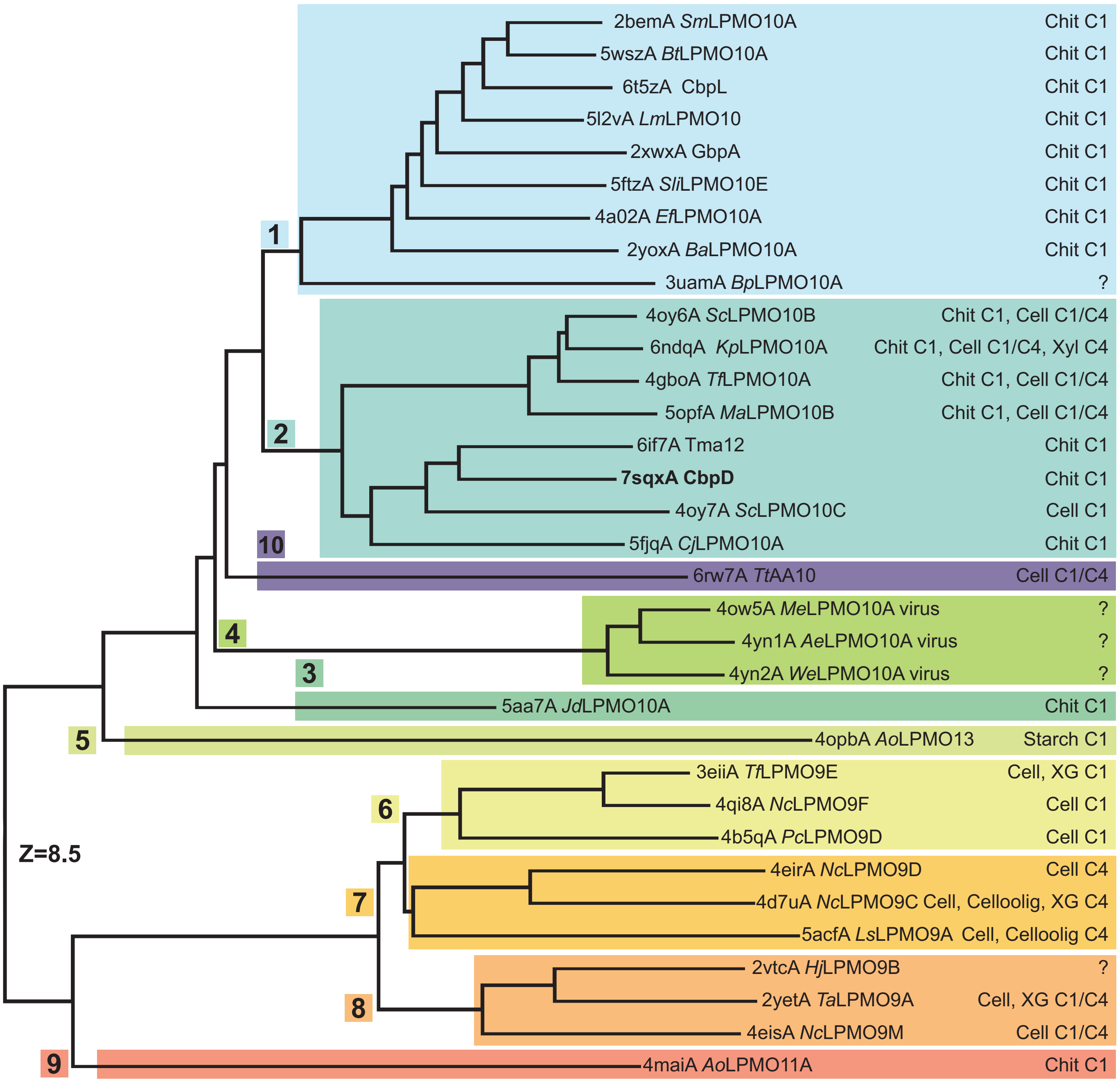
2bemA SmLPMO10A Chit C1
5wszA BtLPMO10A Chit C1
6t5zA CbpL Chit C1
5l2vA LmLPMO10 Chit C1
2xwxA GbpA Chit C1
5ftzA SliLPMO10E Chit C1
4a02A EfLPMO10A Chit C1
2yoxA BaLPMO10A Chit C1
3uamA BpLPMO10A ?
4oy6A ScLPMO10B Chit C1, Cell C1/C4
6ndqA KpLPMO10A Chit C1, Cell C1/C4, Xyl C4
4gboA TfLPMO10A Chit C1, Cell C1/C4
5opfA MaLPMO10B Chit C1, Cell C1/C4
6if7A Tma12 Chit C1
7sqxA CbpD Chit C1
4oy7A ScLPMO10C Cell C1
5fjqA CjLPMO10A Chit C1
6rw7A TtAA10 Cell C1/C4
4ow5A MeLPMO10A virus ?
4yn1A AeLPMO10A virus ?
4yn2A WeLPMO10A virus ?
5aa7A JdLPMO10A Chit C1
4opbA AoLPMO13 Starch C1
3eiiA TfLPMO9E Cell, XG C1
4qi8A NcLPMO9F Cell C1
4b5qA PcLPMO9D Cell C1
4eirA NcLPMO9D Cell C4
4d7uA NcLPMO9C Cell, Celloolig, XG C4
5acfA LsLPMO9A Cell, Celloolig C4
2vtcA HjLPMO9B ?
2yetA TaLPMO9A Cell, XG C1/C4
4eisA NcLPMO9M Cell C1/C4
4maiA AoLPMO11A Chit C1
1
2
10
4
3
5
6
7
8
9
Z=8.5

Figure S2 The structural diversity of LPMOs: The expanded dendrogram of unique LPMO crystal structures from Vaaje-Kolstad *et al.* 2017 in addition to CbpD (bold) and subsequent LMPOAA10 structures determined and listed in the CAZy AA10 database. CbpD is a member of structural cluster 2, and *Tt*AA10 may represent a possible 10th structural cluster. Structures are identified by their PDB identifier and the chain ID, followed by their known substrate(s). The scale indicates the DALI Z-score. Some LPMOs have been referred to by other names in the literature, which are indicated in parenthesis; *Sm*LPMO10A (CBP21), CbpL (*Pl*LPMO10A), GbpA (*Vc*LPMO10B, VcAA10B), *Ef*LPMO10A (EfCBM33A, EfaCBM33), *Ba*LPMO10A (BaAA10A, ChbB, BaCBM33), *Tf*LPMO10A (E7), *Sc*LPMO10C (CelS2, ScAA10C), *Ao*LPMO13 (Ao(AA13)), *Tt*LPMO9E (TtGH61E), *Pc*LPMO9D (PcGH61D), *Nc*LPMO9D (PMO-2, NCU01050), *Nc*LPMO9C (NCU02916), *Ls*LPMO9A (Ls(AA9)A), *Hj*LPMO9B (EG7, Cel61B), *Ta*LPMO9A (TaGH61A), *Nc*LPMO9M (PMO-3, NCU07898), *Ao*LPMO11 (Ao(AA11)). Following the naming convention of subsequently discovered and characterized LPMOs, CbpD may also be referred to as *Pa*LPMO10A, Tma12 may also be named *Tm*LPMO10A, and TtAA10 may be called *Tt*LPMO10A.

The organism of each LPMO is:
Serratia marcescens (*Sm*LPMO10A); *Bacillus thuringiensis* Serovar *kurstaki* (*Bt*LPMO10A); *Photorhabdus luminescens* (CbpL)*; Listeria monocytogenes* (*Lm*LPMO10)*; Vibrio cholera* (GpbA)*; Streptomyces lividans* (*Sli*LPMO10E)*; Enterococcus faecalis* (*Ef*AA10A); *Bacillus amyloliquefaciens* (*Ba*AA10A); *Burkholderia pseudomallei* (*Bp*AA10A)*; Streptomyces coelicolor* (*Sc*LPMO10B, *Sc*LPMO10C)*; Kitasatospora papulose* (*Kp*LPMO10A)*; Thermobifida fusca* (*Tf*LPMO10A)*; Micromonospora aurantiaca* (*Ma*LPMO10B)*; Tectaria macrodonta* (Tma12)*; Pseudomonas aeruginosa* (CbpD); *Cellvibrio japonicus* (*Cj*LPMO10A)*; Teredinibacter turnerae* (*Tt*AA10A); unidentified entomopoxvirus (*Me*LPMO10A, *We*LPMO10A)*; Anomala cuprea entomopoxvirus* (*Ae*LPMO10A)*; Jonesia denitrificans* (*Jd*LPMO10A); *Aspergillus oryzae* (*Ao*LPMO13, *Ao*LPMO11A); *Thermothielavioides terrestris* (*Tf*LPMO9E)*; Neurospora crassa* (*Nc*LPMO9F, *Nc*LPMO9D, *Nc*LPMO9C, *Nc*LPMO9M)*; Phanerodontia chrysosporium* (*Pc*LPMO9D)*; Lentinus similis* (*Ls*LPMO9A); *Trichoderma reesei* (*Hj*LPMO9B)*; Thermoascus aurantiacus* (*Ta*LPMO9A)

LPMO	160 … 169	Substrate
Pseudomonas aeruginosa **CbpD**	TGKHVIYNVWQR	Chitin
Bacillus amyloliquefaciens ***Ba*LPMO10A**	SGYHIILGVWDV	Chitin
Tectaria macrodonta **Tma12**	SGSHLIYVIWQR	Chitin
Photorhabdus laumondii **CbpL**	QGYHVILGVWTI	Chitin
Cellvibrio japonicus ***Cj*LPMO10A**	TGRHIIYSIWQR	Chitin
Enterococcus faecalis ***Ef*LPMO10A**	KGYHVIYAVWGI	Chitin
Jonesia denitrificans ***Jd*LPMO10A**	TGEHTILARWNV	Chitin
Listeria monocytogenes ***Lm*LPMO10**	SGYYLILGVWNI	Chitin
Serratia marcescens BJL200 ***Sm*LPMO10A**	SGSHVILAVWDI	Chitin
Streptomyces ambofaciens ***Sam*LPMO10B**	DGRQKVLAVWNV	Chitin
Streptomyces griseus ***Sg*LPMO10F**	TGKQKVLAVWNV	Chitin
Streptomyces lividans ***Sli*LPMO10E**	SGHHVILAVWTV	Chitin
Vibrio cholerae **GbpA**	EGYQVILAVWDV	Chitin
Bacillus thuringiensis ACCC10066 ***Bt*LPMO10A**	SGYHLILAVWEI	Chitin
Bacillus licheniformis ***Bl*AA10A**	LGYHVILAVWDV	Chitin
Bacillus thuringiensis ATCC33679 ***Bt*LPMO10A-FL**	SGYHVILAVWDV	Chitin
Bacillus cereus ***Bc*LPMO10A**	SGYHVILAVWDV	Chitin
Serratia marcescens KCTC2172 **22kDa Protein**	AVRSDPLPCGDI	Chitin
Streptomyces coelicolor ***Sc*LPMO10B**	TGRHVVYTIWQA	Chitin, Cellulose
Kitasatospora papulosa ***Kp*LPMO10A**	SGRHVVYTIWQA	Chitin, Cellulose, Xylose
Thermobifida fusca ***Tf*LPMO10A**	SGRHVVFTIWKA	Chitin, Cellulose
Micromonospora aurantiaca ***Ma*LPMO10B**	TGRHVVYTIWQA	Chitin, Cellulose
Streptomyces coelicolor ***Sc*LPMO10C**	SGDALIFMQWVR	Cellulose

Figure S3 Position 169 in chitin-active sequences: While previous studies have identified that a short, aliphatic residue is conserved in chitin-active LPMOAA10s (residue 169 in CbpD), creating a shallow cavity on the substrate binding surface that could either accommodate the oxygen species cosubstrate or the N-acetyl group of chitin, the sequence alignment of the chitin-active chitin-active LPMOAA10s listed as characterized in the CAZy database, in addition to CbpD and the other members of the LPMO structural cluster 2 shows Arg (red boxes) is present in 16.7% of chitin-active characterized LPMOAA10s. Position numbering is based on the sequence of CbpD. Boxed LPMO names are members of the LPMO structural cluster 2 (Figure S4). The characterized substrate for each LPMOAA10 is provided to the right of the sequence alignment.

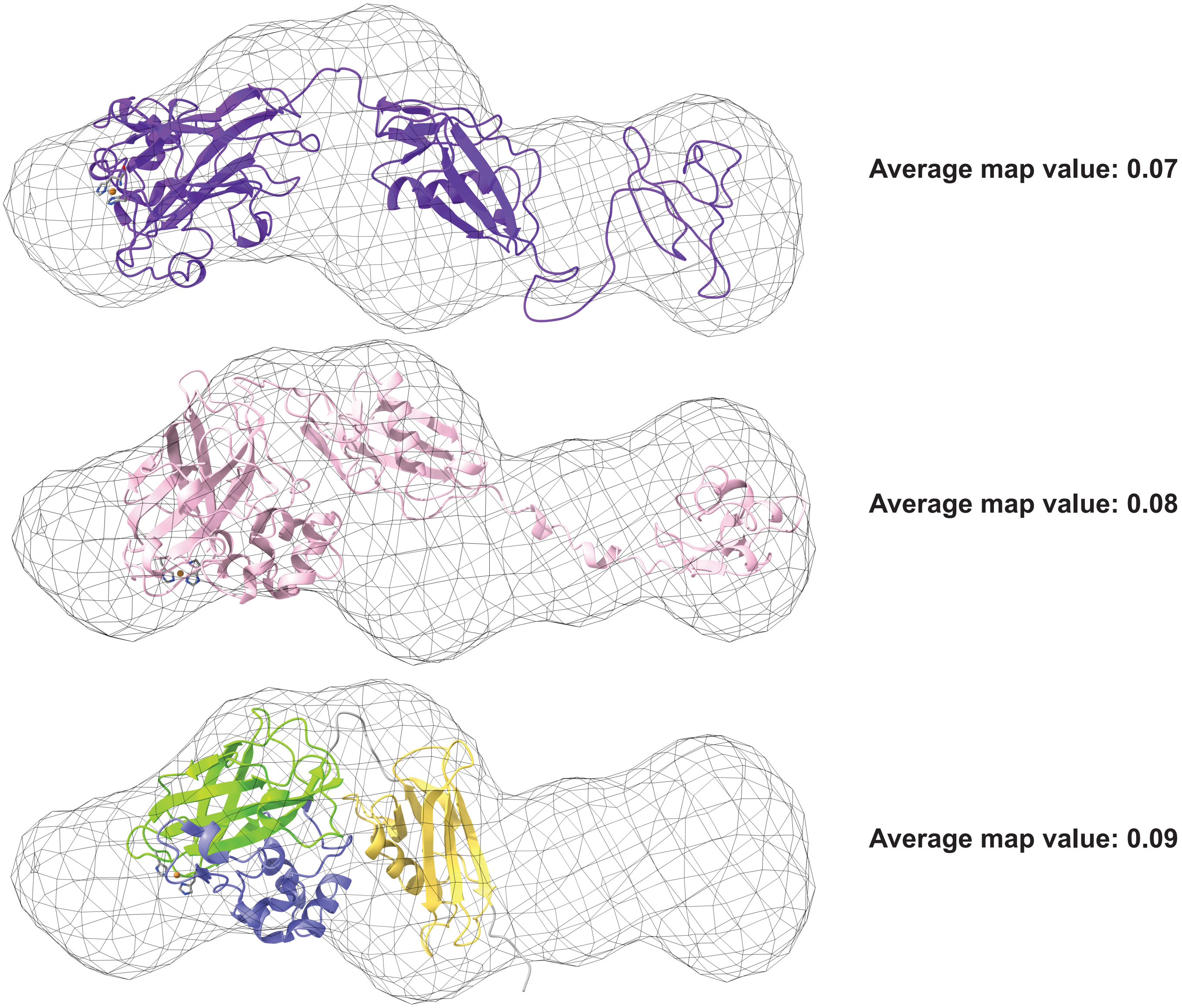

Figure S4: The crystal structure of CbpD adopts a more compact conformation than either observed in solution or predicted by AI: Pepsi-SAXS model (Askarian *et al.*, 2021) (SASBDB ID: SASDK42) (top, purple); RoseTTAFold model (middle, pink); and crystal structure (bottom, colored as in Figure2A) of CbpD fit into the 15 Å resolution volume map of the CbpD SAXS model envelope generated by Askarian *et al.* (2021). Average map values after fitting each structure into the density map are provided (right). His brace (sticks) and a Cu ion (sphere) placed in the active site are shown for orientation.
